# Variations in methane emissions from dairy cows: associations with rumen microbial synergy and metabolic pathway divergence

**DOI:** 10.1186/s40104-026-01432-9

**Published:** 2026-06-15

**Authors:** Peng Jia, Lifeng Dong, Tao Ma, Yanliang Bi, Yan Tu, Qiyu Diao

**Affiliations:** 1 Institute of Feed Research, Chinese Academy of Agricultural Sciences/Sino-US Joint Lab on Nutrition and Metabolism of Ruminant/Key Laboratory of Feed Biotechnology of the Ministry of Agriculture and Rural Affairs, Beijing, 100081 People’s Republic of China; 2 Institute of Animal Husbandry and Veterinary Science, Shanghai Academy of Agricultural Sciences/Key Laboratory of Livestock and Poultry Resources (Pig) Evaluation and Utilization of Ministry of Agriculture and Rural Affairs/Shanghai Engineering Research Center of Breeding Pig, Shanghai, 201106 People’s Republic of China

**Keywords:** Dairy cows, Methane, Methane mitigation, Rumen methanogenesis, Rumen microbiota

## Abstract

**Background:**

Methane (CH_4_) is a metabolic by-product of rumen microbial fermentation, contributing significantly to global warming and dietary energy loss. Elucidating the mechanisms underlying natural variation in rumen methanogenesis is essential for the development of effective CH_4_ mitigation strategies. Here, we applied rumen metagenomics to identify the microbial mechanisms for differences in enteric CH_4_ emissions among dairy cows.

**Results:**

Enteric CH_4_ emissions from 111 lactating dairy cows under normal feeding conditions were utilized to characterize the natural variation in rumen methanogenesis. Metagenomic analysis revealed that the comprehensive effects of bacteria involved in starch degradation, lactate metabolism, and volatile fatty acid biosynthesis provide distinct amounts of hydrogen for rumen methanogenesis in high-methane-producing (HMP) and low-methane-producing (LMP) cows. Ciliate protozoa were universally abundant in HMP cows (*P* < 0.05), whereas methanogens enrichment exhibited heterogeneity, with the dominant methanogen *Methanobrevibacter* exhibiting negative correlations with the other 11 methanogens (*P* < 0.05). Six nutrient metabolic pathways modulating methanogenesis were identified, and HMP-associated methanogenesis was further driven by upregulated formate metabolism and acetoclastic pathways (*P* < 0.05). Random forest model analysis screened 34 microbial genera as biomarkers for CH_4_ production.

**Conclusions:**

This study excluded extrinsic confounders exist for rumen microbiome and CH_4_ emissions in dairy cows. These findings elucidated the causal microbial and metabolic mechanisms underlying rumen methanogenesis, providing actionable targets for microbiome-based strategies to mitigate CH_4_ emissions from livestock farming.

**Supplementary Information:**

The online version contains supplementary material available at 10.1186/s40104-026-01432-9.

## Background

The melting of glaciers is accelerated by global warming, which simultaneously raises the frequency and intensity of extreme weather events and damages ecosystems. As we know, anthropogenic emissions of greenhouse gases (GHGs) play a crucial role in driving global warming [1]. Among GHGs, methane (CH_4_) has a short atmospheric lifetime and high global warming potential, making its reduction a rapid means to mitigate global warming [2, 3]. Furthermore, this reduction constitutes a necessary condition for limiting the temperature rise to 1.5 °C above pre-industrial levels [4]. Consequently, the reduction of CH_4_ emissions has attracted increasing attention, with more than 110 countries and supporters signing the Global Methane Pledge. Deciphering the sources and mechanisms of CH_4_ production and proposing new procedures to reduce CH_4_ emissions is a growingly prominent international topic [5].

Enteric CH_4_ from ruminants accounts for 30% of global anthropogenic CH_4_ emissions [2], with dairy cattle contributing approximately 30% of total livestock emissions [6]. Dairy cows provide nearly 80% of the milk consumed by humans [7], a nutrient-rich source containing amino acids, bioactive fatty acids, and essential micronutrients [8, 9]. It is notable that CH_4_ emitted by cows means the loss of dietary energy and will subsequently reduce milk yield. Additionally, CH_4_ adds to the GHG in the atmosphere that contribute to global warming [10]. Although some dietary interventions can mitigate CH_4_ emissions, the effect of inhibiting rumen methanogenesis may be transitory [11]. In some cases, CH_4_ emissions were returned to baseline levels when the supply of CH_4_ inhibitors to ruminants was discontinued [12]. Furthermore, the reversal of inhibiting rumen methanogenesis after offering additives for a longer period may be attributed to the adaptation of the rumen microorganisms to these additives [12]. Some previous studies reported that rumen microorganisms, especially methanogens, also did not respond instantaneously to inhibitors [11]. Pitta et al. [13] indicated that methanogens have inconsistent response to 3-nitrooxypropanol, currently one of the most effective CH_4_ inhibitors. Although CH_4_ is produced directly by methanogens, it is also dependent on bacteria and protozoa that produce hydrogen (H_2_) and carbon dioxide (CO_2_) [14]. Therefore, elucidating the aspects and details of the interactions with ruminant physiology and rumen microorganisms in methanogenesis remains a critical research priority.

How to mitigate global warming and dietary energy loss associated with ruminant CH_4_ emissions depends on our understanding of the mechanisms of rumen methanogenesis. Rumen methanogenesis is a highly complex and multifactorial trait, and while several studies have been published on rumen fermentation and CH_4_ production from biochemistry [15] and microbiology [16, 17], substantial gaps remain. A more comprehensive elucidation of the enzymes and genes involved in the metabolic pathways associated with rumen methanogenesis, as well as the key microorganisms involved in CH_4_ formation, is essential. The advent of high-throughput technology enables the exploration of the complicated relationship between the ruminal microbiome and its function in rumen methanogenesis in large herds of ruminants [18, 19]. Although accurately measuring CH_4_ emissions from ruminants under normal feeding conditions is challenging, we were fortunate to successfully measure the CH_4_ emissions from 111 lactating Holstein dairy cows housed in the same barn using the GreenFeed system [20]. Based on these data, it is necessary to study rumen microbial population and functional characteristics associated with natural variation of CH_4_ emissions in dairy cows. In this study, we conducted rumen metagenomics on dairy cows with notably varied CH_4_ production to investigate the following queries: Under the same feeding conditions, (1) What are the characteristics of microorganisms in high-CH_4_-emissions dairy cows and their interactions on methanogenesis? (2) What are the differences in methane metabolism and nutrient metabolism pathways in dairy cows with different CH_4_ production? (3) Which ruminal microbial characteristics can be used as predictive biomarkers of CH_4_ production? The objective of this study is to uncover new biological rules and characteristics that will help us to further explain the key pathways and mechanisms of rumen methanogenesis, thereby clarifying strategies that can practically mitigate CH_4_ emissions from dairy cows. The whole results could provide valuable information for the development of a more environmentally friendly dairy industry.

## Methods

### Cows and samples

This study was carried out at a commercial dairy farm called the Yinxiangweiye International Third Farm in Shandong Province, China. The experimental protocol for this study was approved by the Institute of Feed Research of Chinese Academy of Agricultural Sciences (IFR-CAAS-20191018). The same method was used to manage the experimental cows, which were fed in a freestall barn with the same diet. Physiological parameters and CH_4_ emissions from this herd of Holstein dairy cows (*n* = 111; 2.8 ± 1.0 parity, 138 ± 19 DIM, and 38.1 ± 6.9 kg/d of milk yield) have been measured [20]. CH_4_ emissions of these cows in normal feeding conditions were determined by the GreenFeed unit (C-Lock Inc., South Dakota, USA). Every animal had at least 20 valid visits, with each visit lasting more than 3 min. The experiment lasted 90 d. We selected 9 cows with the highest CH_4_ production (CH_4_ = 403 ± 22.06 g/d) and 9 cows with the lowest CH_4_ production (CH_4_ = 261 ± 22.27 g/d) from this herd, then classified them into two groups: high-methane-production group (HMP), low-methane-production group (LMP) (Table 1).

**Table 1:** Comparison of physiological parameters between low-methane-production and high-methane-production cows

Performance	Group		SEM	*P*-value
	**LMP**	**HMP**		
Methane production, g/d	261.00	403.22	17.976	< 0.001
Methane/ECM, g/kg	8.01	10.82	0.486	0.001
Age, months	50.40	54.92	3.358	0.517
Days in milk, d	134.78	138.56	4.447	0.684
Metabolic weight, kg	129.57	135.93	1.906	0.095
Milk yield, kg/d	35.27	38.86	1.480	0.236
ECM yield, kg/d	33.28	38.01	1.413	0.094
Total VFA, mmol/L	104.18	96.79	2.488	0.142
pH value	5.99	6.07	0.053	0.514
Proportion, %				
Acetate	56.84	62.29	0.902	< 0.001
Propionate	28.15	22.30	0.981	< 0.001
Butyrate	11.01	12.03	0.393	0.202
Valerate	2.33	1.59	0.164	0.019
Acetate/Propionate	2.04	2.85	0.134	< 0.001

Data were analyzed using *t*-tests

*ECM* Energy-corrected milk, *VFA* Volatile fatty acids, *LMP* Low methane production, *HMP* High methane production

On the last day, 2 h after the morning feeding, rumen samples were extracted from every cow using an oral stomach tube [21]. To reduce the contamination of saliva, the initial 150–200 mL rumen contents were discarded and the remaining 100–200 mL rumen contents were collected. The pH values of the rumen contents were immediately measured using a pH meter (model PB-20, Sartorius AG, Goettingen, Germany). Two subsamples of 1 mL of rumen content were snap-frozen in nitrogen liquid immediately for metagenomic analysis. The remainder was filtered through four layers of cheesecloth, and a 10-mL aliquot was collected and stored at −20 °C for subsequent ruminal volatile fatty acid (VFA) analysis [22].

### DNA extraction, metagenome sequencing and data processing

DNA was extracted from rumen contents based on repeated bead-beating plus column method [23]. Agarose gel electrophoresis was used to verify the integrity of the DNA. The quality and quantity of DNA were measured on the NanoDrop 2000 spectrophotometer (Thermo Fisher Scientific, Delaware, USA). TrueSeq DNA PCR-Free Library Preparation Kit (Illumina, California, USA) constructed individual metagenomic libraries from each sample. Metagenome library sequencing (2 × 150 paired-end) was performed using the Illumina HiSeq 3000 platform at Beijing Allwegene Tech Ltd. (Beijing, China).

Sickle was used to perform quality control on the datasets by trimming the 5'- and 3'-ends of reads, removing low-quality bases with quality scores < 20, and discarding reads shorter than 50 bp or containing ambiguous “N” bases. The quality-filtered reads were subsequently aligned to the bovine genome using BWA to remove host DNA sequences [24]. Chloroplast and mitochondrial sequences were also removed, retaining only microbial sequences for downstream analysis [25]. Megahit was used for individual de novo assembly of the filtered reads from each sample [26]. Open reading frames (ORFs) were predicted with MetaGene from assembled contigs longer than 300 bp [27]. Using a sequence identity cut-off of 95%, CD-HIT preserved and clustered the ORFs produced from assembled contigs into a non-redundant data set [28]. Reads from individual sample and assembly results were mapped to nonredundant gene sets to estimate the abundance using SOAPaligner v2.21 [29].

To evaluate the richness and diversity of bacterial and archaeal communities, alpha diversity indices, including Chao1, ACE, and Shannon, were calculated at the OTU level using QIIME (Version 1.9.1) [30]. Rarefaction curves were constructed to evaluate the sequencing saturation of the microbial community. Principal coordinate analysis (PCoA) was performed to assess beta diversity, aiming to reveal compositional differences in species among sample groups. Venn diagrams were generated using the VennDiagram package in R (Version 3.6.3) to visualize the shared and unique OTUs between the two experimental groups. Only OTUs detected in at least 80% of samples within a given group and with a total relative abundance higher than 0.01% were included in the Venn diagram intersection analysis. Using DIAMOND (v0.8.35) [31], the contigs were evaluated taxonomically against the RefSeq database [32]. Taxonomic profiles were analyzed at phylum, genus, and species levels. HUMAnN v2.0 was used to calculate relative abundances for each taxonomic level. Microbial taxa in at least one group that had a relative abundance higher than 0.1% were selected for downstream analysis.

### Bioinformatics and statistical analysis

For functional analysis, contigs with an *E* value of 1 × 10^−5^ were annotated using DIAMOND against the KEGG profiles [33]. Using HMMscan against the CAZy database, CAZymes with an *E* value of 1 × 10^−5^ were annotated [34]. The SparCC program was used to analyse co-occurrences between bacterial or archaeal taxa with default settings. Correlations between microbial taxa and functions (Carbohydrate metabolism and Energy metabolism) were calculated using Spearman correlation analysis. In the co-occurrence and correlation analyses, only microbial taxa with a relative abundance higher than 0.1% at the genus level and those with a correlation coefficient higher than 0.8 or lower than −0.8 and a *P* value lower than 0.05 were utilized in the co-occurrence network analysis. Cytoscape (Version 3.2.1, http://www.cytoscape.org) was used for the visualization of the networks. Based on the Maximal Clique Centrality approach (https://apps.cytoscape.org/apps/cytohubba), the CytoHubba tool in Cytoscape software was utilized to identify the hubs of the microbes in the networks.

Using the randomForest package in R, rumen microbes were input into the random forest model. Ranking the importance of microbes in the model based on the mean decrease accuracy score. The most predictive microbes were found based on the maximum area under the curve using the UC-RF algorithm (REF). To further evaluate the model, a 99-fold cross-validation scheme was applied using the rfUtilities package in R (Version 3.6.3, https://cran.rproject.org/web/packages/rfUtilities/index.html). From the selected microbiomes, a correlation heatmap was generated with rumen fermentation characteristics and CH_4_ production.

### Statistical analysis

The *t*-test procedure in SAS version 9.2 (SAS Institute Inc., Cary, NC, USA) was performed to assess the differences of CH_4_ emissions, ages, days in milk, metabolic weight, milk yield, ECM yield, and rumen fermentation characteristics between the LMP and HMP. Differences in microbial relative abundance and alpha diversity between the LMP and HMP were evaluated using the Wilcoxon rank‑sum test of SPSS (SPSS Inc., Illinois, USA) version 19.0. Beta diversity was examined using principal coordinate analysis (PCoA) based on the Bray–Curtis dissimilarity matrix. The Wilcoxon rank‑sum test of SPSS 19.0 was also used to analyse the abundances of microbial metabolic pathways, modules, KEGG enzymes, and CAZymes between the LMP and HMP groups. The Spearman rank correlation coefficients and tests of significance between rumen microbial taxa and functions were calculated using the SPSS 19.0. Differences were considered significant at *P* < 0.05.

## Results

### Methane emissions and rumen fermentation parameters of dairy cows

In the present experiment, GHG emissions from 111 cows have previously been measured by the GreenFeed system. Based on the CH_4_ emissions, 9 cows with the lowest CH_4_ production (LMP) and 9 cows with the highest CH_4_ production (HMP) were selected respectively for rumen metagenome analyses. The cows were housed in a freestall barn and fed the same diet, with no differences (*P* > 0.05) in age, days in milk, metabolic weight, or milk yield between the LMP and HMP groups (Table 1). Furthermore, rumen pH values and total VFA concentrations did not differ (*P* > 0.05) between the LMP and HMP (Table 1). The differences between the two groups were as follows: Enteric CH_4_ production (g/d) and CH_4_ intensity (g/kg) were higher (*P* < 0.05) in HMP than in the LMP (Table 1). The proportion of acetate was higher (*P* < 0.05) in the HMP, while that of propionate and valerate was lower (*P* < 0.05) in HMP than in the LMP. Consequently, the HMP had a higher (*P* < 0.05) acetate to propionate ratio than the LMP (Table 1).

### Metagenomic data statistics, rumen VFA and methane metabolism

Metagenome sequencing generated a total of 839,749,131 reads, with 46,652,730 ± 2,617,805 reads (mean ± SD) per sample. The rumen metagenome consisted of 44.29% bacteria (744,050,389 sequences), 0.71% archaea (11,962,024 sequences), 0.19% eukaryota (3,260,168 sequences), 0.11% virus (1,778,230 sequences), 0.03% fungi (448,751 sequences), and 54.67% unidentified sequences (917,998,700 sequences). The microbial domains between the rumen microorganisms of the two groups were compared, and there were significant differences in eukaryota and virus between the two groups (adjusted *P* < 0.05, Fig. S1), such as Ciliophora exhibited a higher relative abundance in the HMP group than in the LMP group (*P* < 0.05, Table S1).

**Fig. 1:**
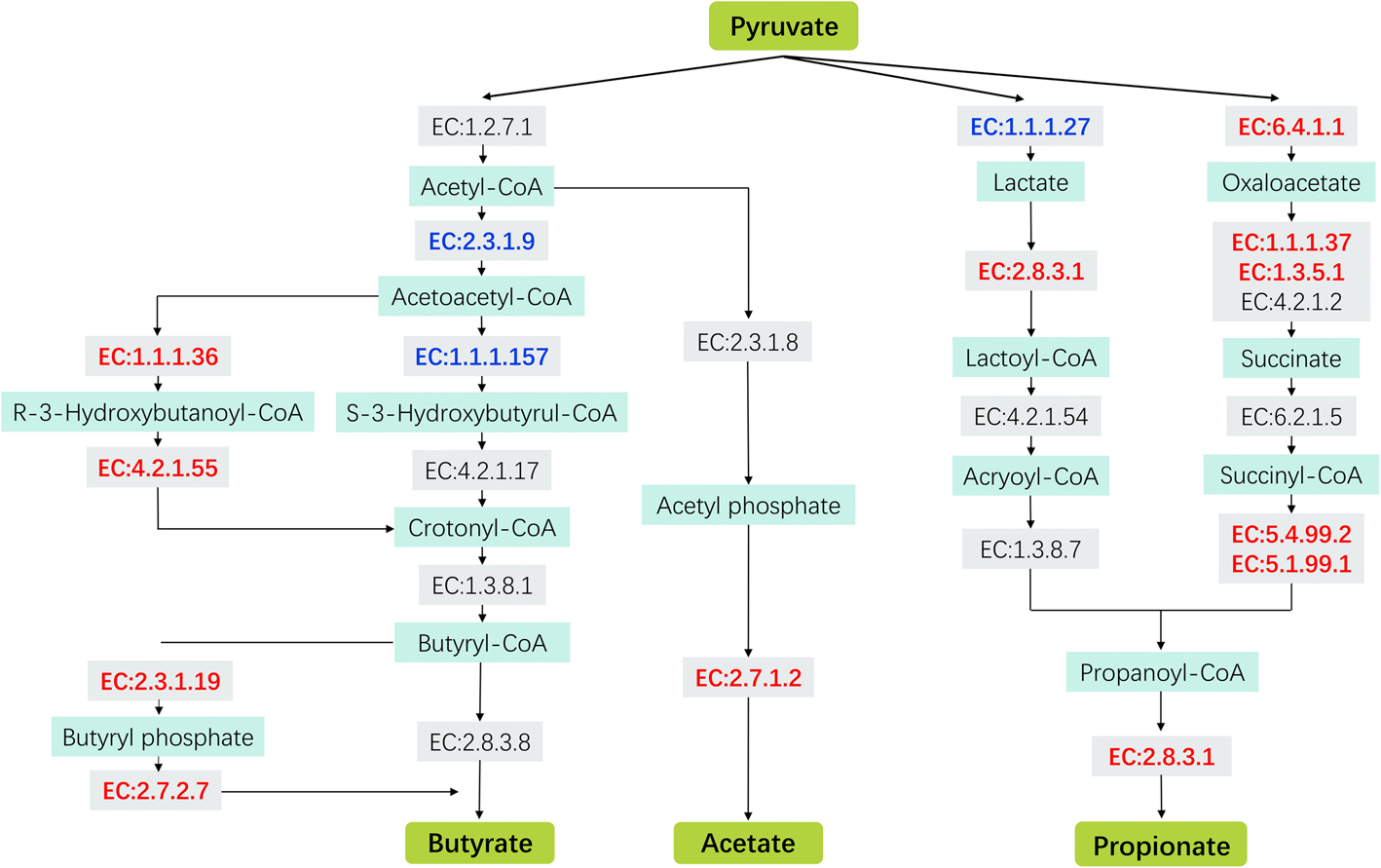
Metabolic routes for butyrate, acetate, and propionate production in rumen. The Wilcoxon rank‑sum test was used to compare means, with *P* value < 0.05 indicating a significant difference. Red text indicated significantly up-regulated KO enzymes, whereas blue text indicated significantly down-regulated KO enzymes in the HMP cows

**Fig. 2:**
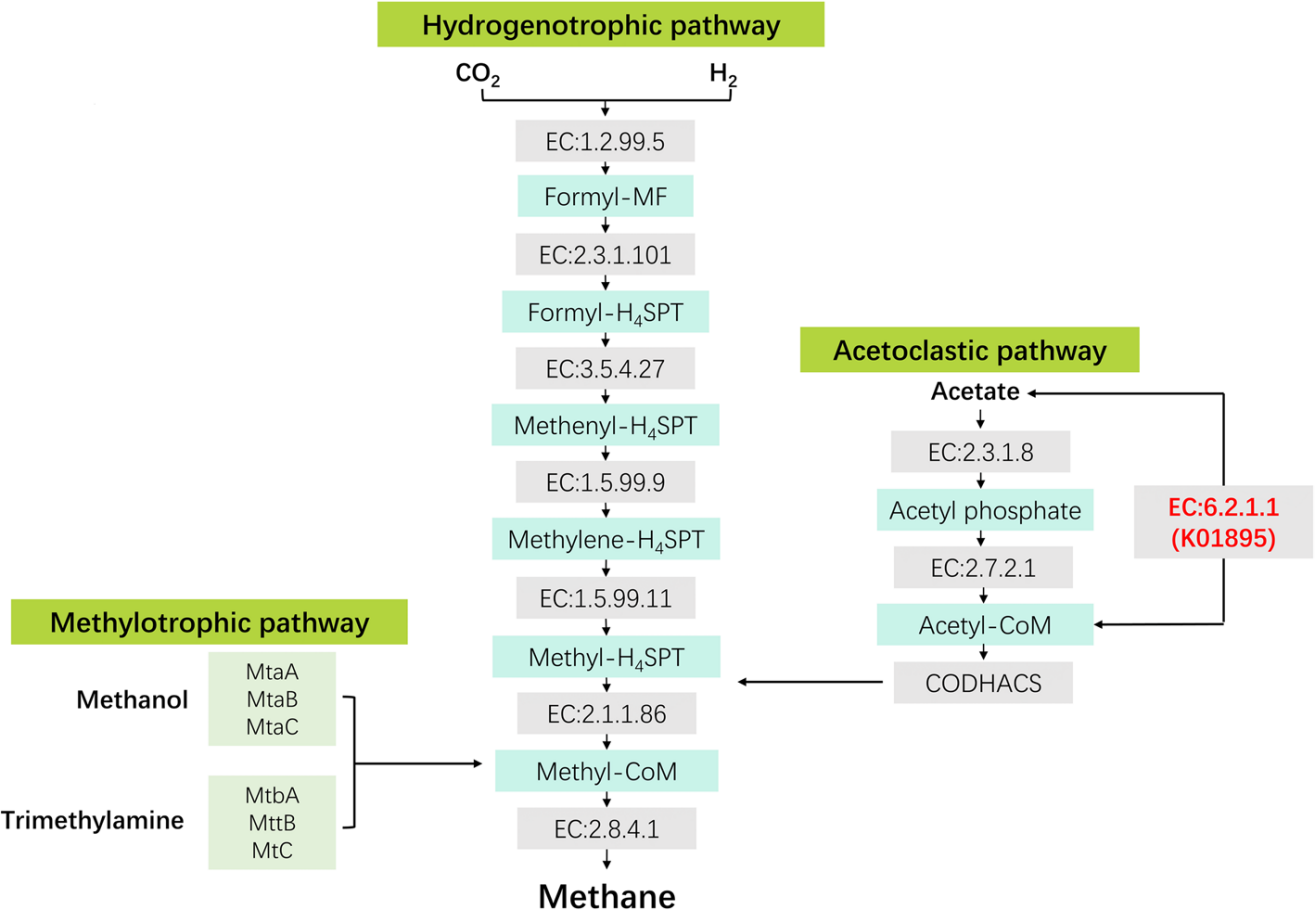
Metabolic pathways of rumen methanogenesis in cows. The Wilcoxon rank‑sum test was used to compare means, with *P* value < 0.05 indicating a significant difference. Red text indicated significantly up-regulated enzyme genes in the HMP cows

Differences in key enzymes determine the specificity of CH_4_ production. The production of acetate and butyrate release H_2_, which can become a source of energy for methanogens. Propionate production, however, can reduce enteric CH_4_ emissions due to it compete with methanogens for H_2_. As shown in Fig. 1, we screened 34 encoding enzymes involved in the fermentation pathway for the metabolism of glucose to acetate, propionate, and butyrate. (1) From glucose to pyruvate, 1 enzyme was enriched (*P* < 0.05) in HMP, whereas 3 enzymes were enriched (*P* < 0.05) in the LMP. (2) Focusing on acetate and butyrate production, 5 enzymes were enriched (*P* < 0.05) in HMP, compared to only 2 enzymes (*P* < 0.05) in the LMP. (3) In the propionate formation, 7 enzymes were enriched (*P* < 0.05) in HMP. Additionally, 1 enzyme (EC:1.1.1.27) involved in the acrylate route of propionate production was enriched (*P* < 0.05) in the LMP. (4) We further focused on the methane metabolism. Acetyl-CoA synthetase (EC:6.2.1.1) and its corresponding gene K01895 were more abundant in the HMP, and both were involved in the acetoclastic pathway of methanogenesis according to the KEGG database (Fig. 2, Tables S2 and S3).

###  Taxonomic configuration of ruminal bacteria is the important factor affecting CH_4_ emissions

**Fig. 3:**
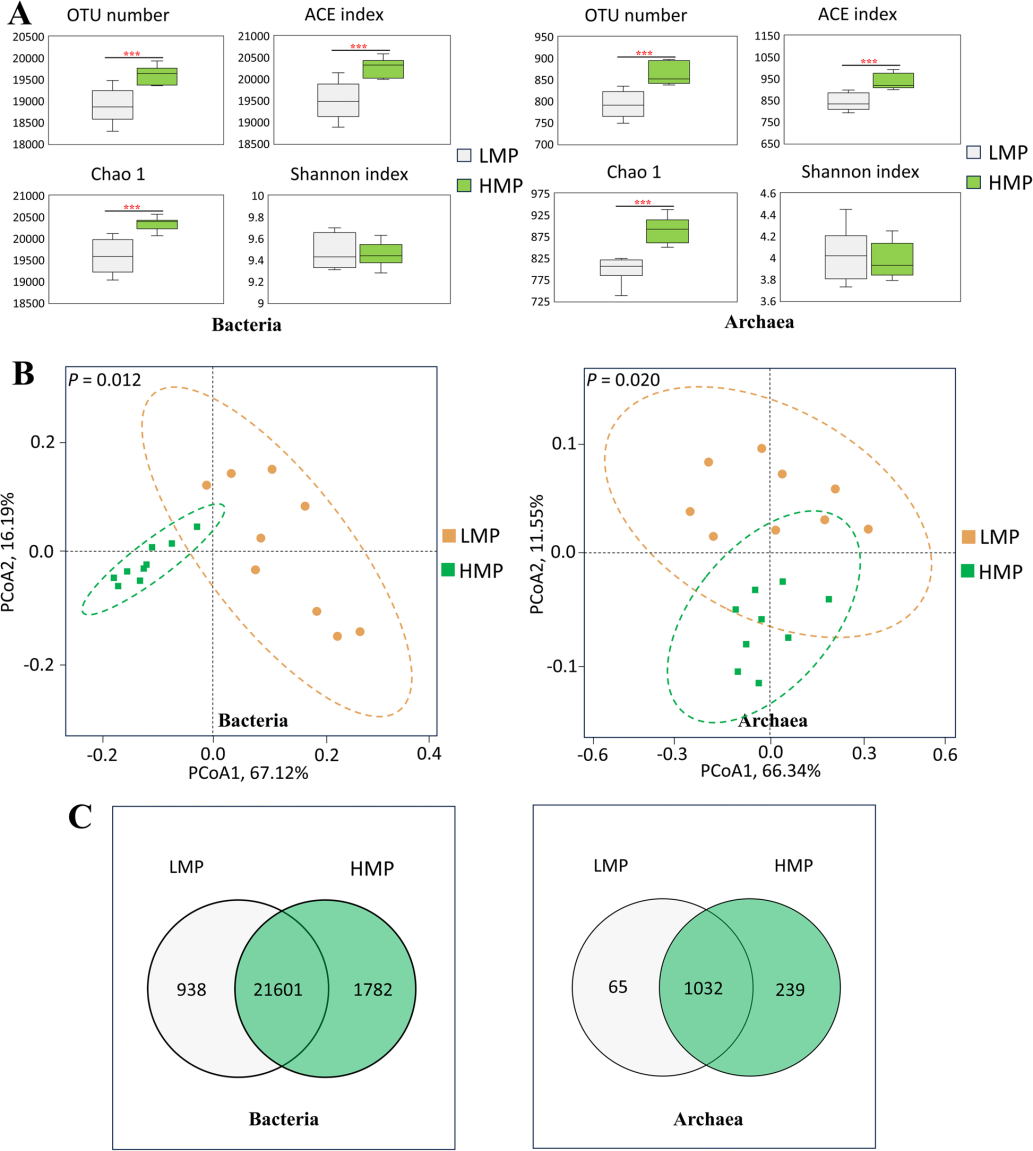
Diversity of rumen microbial communities of cows. **A** OTU number and α-diversity indexes. **B** PCoA plot of ruminal bacterial and archaeal diversity. **C** Venn diagram of OTUs. The Wilcoxon rank‑sum test was used to compare means, with *P* value < 0.05 indicating a significant difference. ^*^P < 0.05, ^**^P < 0.01, ^***^P < 0.001

Compared with the LMP group, the HMP group exhibited a higher (*P* < 0.05) OTU number, along with greater ACE and Chao indexes; but Shannon indexes were roughly the same between the two groups (*P* > 0.05) (Fig. 3A; Table S4). The PCoA plot revealed a clear separation between the LMP and HMP on basis of the bacterial species (Fig. 3B). In addition, Venn profile of bacteria showed that 21,601 OTUs were shared between the LMP and HMP; while the HMP had a higher number of unique OTUs than the LMP (1,782 vs. 938; Fig. 3C).

**Fig. 4:**
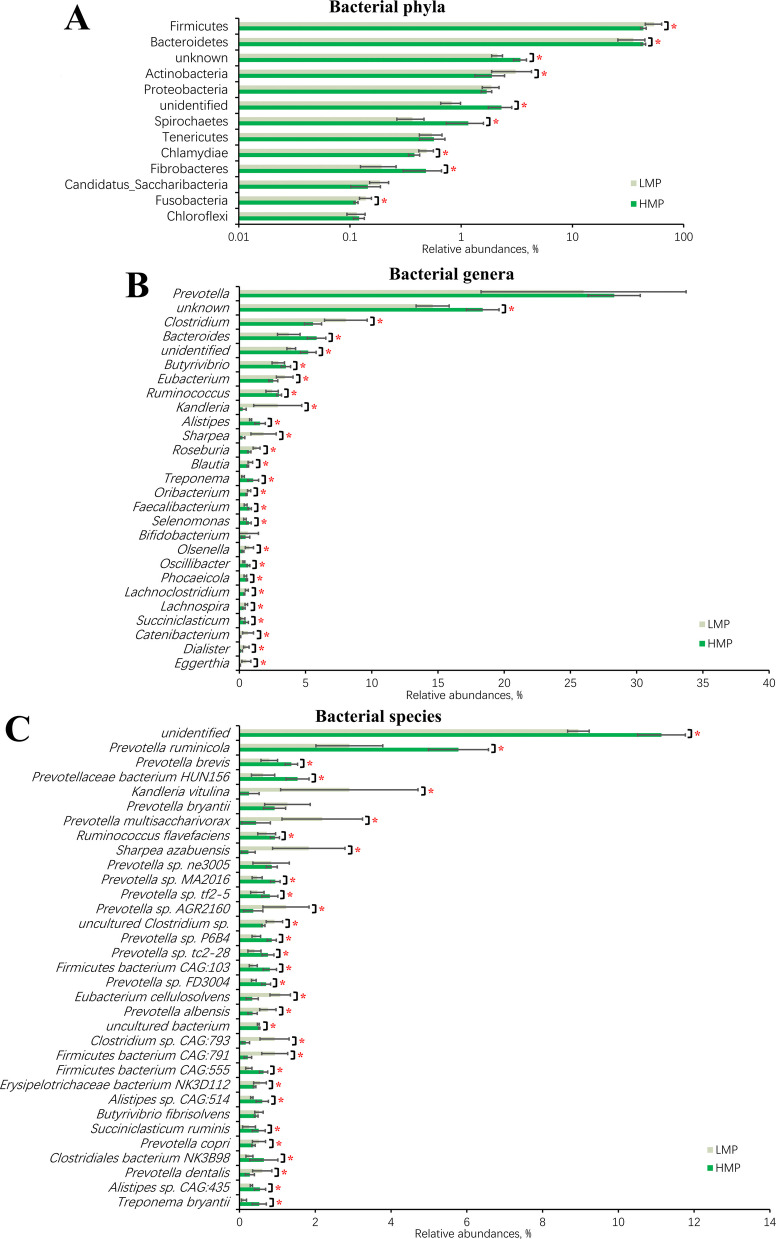
The relative abundances of bacteria in rumen. Relative abundances of bacterial communities at the phylum (**A**), genus (**B**), and species (**C**) levels. The Wilcoxon rank‑sum test was used to compare means. ^*^P < 0.05

We analyzed the bacterial metagenomic sequences at the phylum, genus, and species levels: (1) Seven phyla varied significantly in relative abundances between the LMP and HMP. Bacteroidetes, Spirochaetes, and Fibrobacteres were more abundant (*P* < 0.05), while the other 4 phyla (Firmicutes, Actinobacteria, Chlamydiae, Fusobacteria) were less abundant (*P* < 0.05) in the HMP than in the LMP (Fig. 4A and Table S1). (2) At the genus level, the 3 most dominant bacteria were *Prevotella*, *Clostridium*, and *Bacteroides*. Twenty-three genera shifted significantly between the two groups, with 10 genera being more (*P* < 0.05) abundant and 13 genera less (*P* < 0.05) abundant in the HMP than in the LMP (Fig. 4B and Table S1). (3) At the species level, the 6 most dominant bacteria were *Prevotella ruminicola*, *Kandleria vitulina*, *Prevotella multisaccharivorax*, *Prevotella bryantii*, *Prevotella brevis*, and *Prevotellaceae bacterium *HUN156. Twenty-nine species showed significant differences in enrichment between the LMP and HMP, the HMP group had 17 species with higher (*P* < 0.05) relative abundance and 12 species with lower relative abundance (Fig. 4C, Table S1).

### Taxonomic configuration of ruminal archaea is another important factor affecting CH_4_ emissions

Similar to the results of total bacteria, the HMP group had greater (*P* < 0.05) OTU number, ACE, and Chao indexes compared with the LMP group; whereas the Shannon indexes remained unchanged (*P* > 0.05) (Fig. 3A and Table S4). PCoA plot revealed a clear segregation between the LMP and HMP on basis of the archaeal species (Fig. 3B). Additionally, the Venn profile of archaea showed that 1,032 OTUs were shared between the LMP and HMP; while the HMP had a higher number of unique OTUs than the LMP (239 vs. 65; Fig. 3C).

**Fig. 5:**
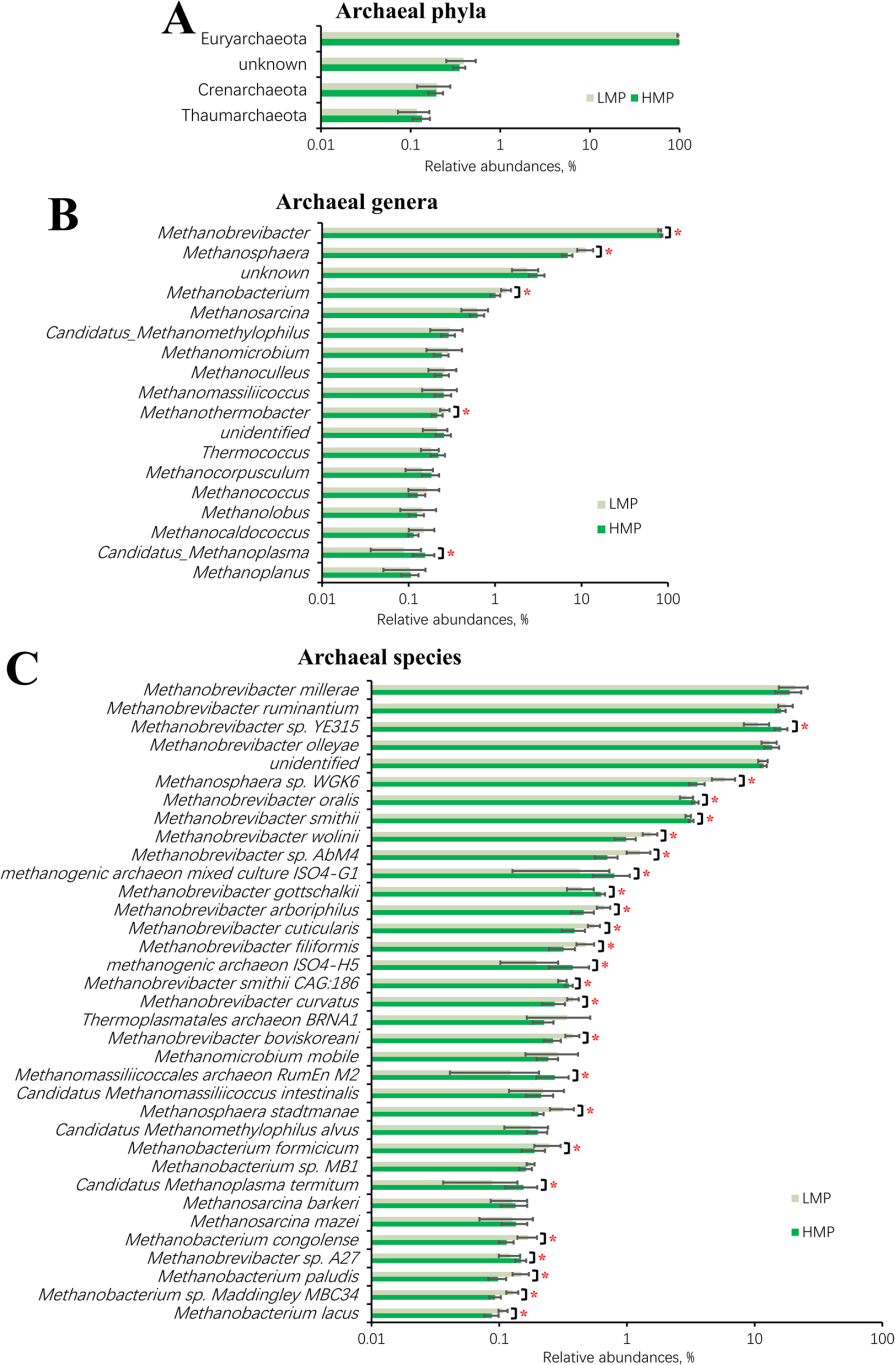
The relative abundances of archaea in rumen. Relative abundances of archaeal communities at the phylum (**A**), genus (**B**), and species (**C**) levels. The Wilcoxon rank‑sum test was used to compare means. ^*^P < 0.05

Archaeal metagenomic sequences were then analyzed at three levels: (1) At the phylum level, no significant difference was observed in the relative abundance of archaea between the LMP and HMP (*P* > 0.05) (Fig. 5A and Table S1). (2) At the genus level, 5 genera shifted significantly during this study. The relative abundances of *Methanobrevibacter* and *Candidatus Methanoplasma* were higher (*P* < 0.05) in the HMP, while those of *Methanosphaera*, *Methanobacterium*, and *Methanothermobacter* were higher (*P* < 0.05) in the LMP (Fig. 5B and Table S1). (3) At the species level, 10 species were more (*P* < 0.05) abundant in the HMP, while 14 species were more (*P* < 0.05) abundant in the LMP (Fig. 5C and Table S1).

### Nutrient metabolic pathways can directly affect CH_4_ emission

**Fig. 6:**
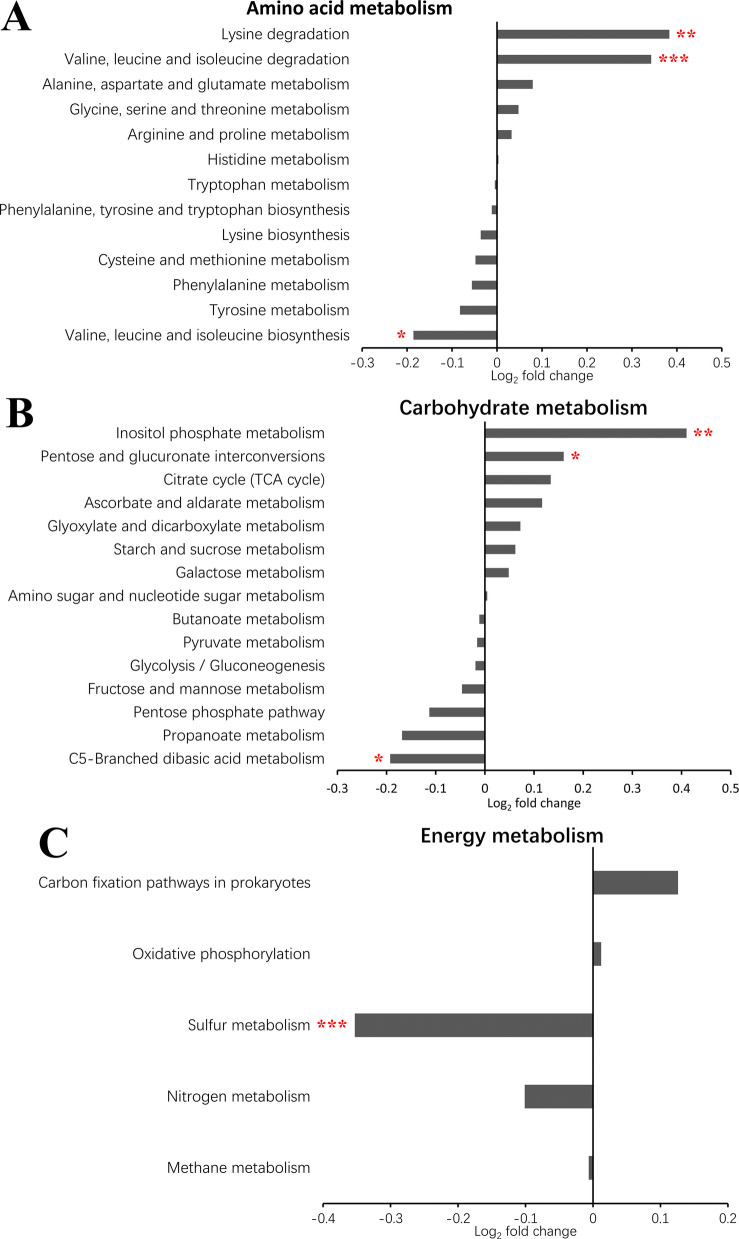
Fold changes of amino acid metabolism (**A**), carbohydrate metabolism (**B**), and energy metabolism (**C**) pathways identified in the metagenomes of the cows. The Wilcoxon rank‑sum test was used to compare means. ^*^P < 0.05, ^**^P < 0.01, ^***^P < 0.001

A total of 390 Kyoto Encyclopedia of Genes and Genomes (KEGG) third-level pathways (cpm ≥ 5 and prevalence ≥ 20%) were revealed according to metagenomic sequences (Table S5). These pathways were divided into 4 first-level categories: “Cellular processes” (4.56%), “Environmental information processing” (6.27%), “Genetic information processing” (16.45%), and “Metabolism” (72.71%). There were 25 s-level categories observed, with the 5 most abundant being “Carbohydrate metabolism” (17.82%), “Global and overview maps” (11.98%), “Amino acid metabolism” (10.18%), “Replication and repair” (8.81%), and “Nucleotide metabolism” (8.72%). Key metabolic pathways for amino acids, carbohydrates, and energy are shown in Fig. 6A–C. Notably, the “valine, leucine and isoleucine degradation”, “lysine degradation”, “pentose and glucuronate interconversions”, and “inositol phosphate metabolism” were enriched (*P* < 0.05) in the HMP. In contrast, 3 pathways (“valine, leucine and isoleucine biosynthesis”, “C5-branched dibasic acid metabolism”, “sulfur metabolism”) were enriched (*P* < 0.05) in the LMP.

There were 228 genes classified into CAZymes (Table S6). Among the genes encoding CAZymes involved in the breakdown of carbohydrates (cellulose, hemicellulose, starch, protein, and lignin), 63 exhibited higher (*P* < 0.05) abundance in the HMP, compared with 20 in the LMP (Fig. S2A). In carbohydrate-binding modules (CBMs) of non-catalytic CAZymes involved in complex carbohydrate deconstruction, 32 showed greater (*P* < 0.05) abundance in the HMP versus only 5 in the LMP (Fig. S2B). Among the glycosyltransferases (GTs) involved in carbohydrate synthesis, 18 were more (*P* < 0.05) abundant in the HMP, while 14 were more (*P* < 0.05) abundant in the LMP (Fig. S2C).

### Microbial interactions effect the level of CH_4_ emissions from dairy cows

The co-occurrence network analysis of rumen bacteria showed 1,335 co-occurrence interactions, with different patterns observed in the LMP and HMP groups. A total of 339 interactions were detected in the HMP cows; the most positive (*P* < 0.05) interactions were found within the Firmicute taxa, while the most negative (*P* < 0.05) interactions were found between the Firmicute and Bacteroidetes taxa (Fig. S3). In addition, 996 interactions were detected in the LMP cows. Similar to the results of HMP cows, the most positive (*P* < 0.05) interactions were found within the Firmicute taxa, while the most negative (*P* < 0.05) interactions were found between the Firmicutes and Bacteroidetes taxa (Fig. S3).

**Fig. 7:**
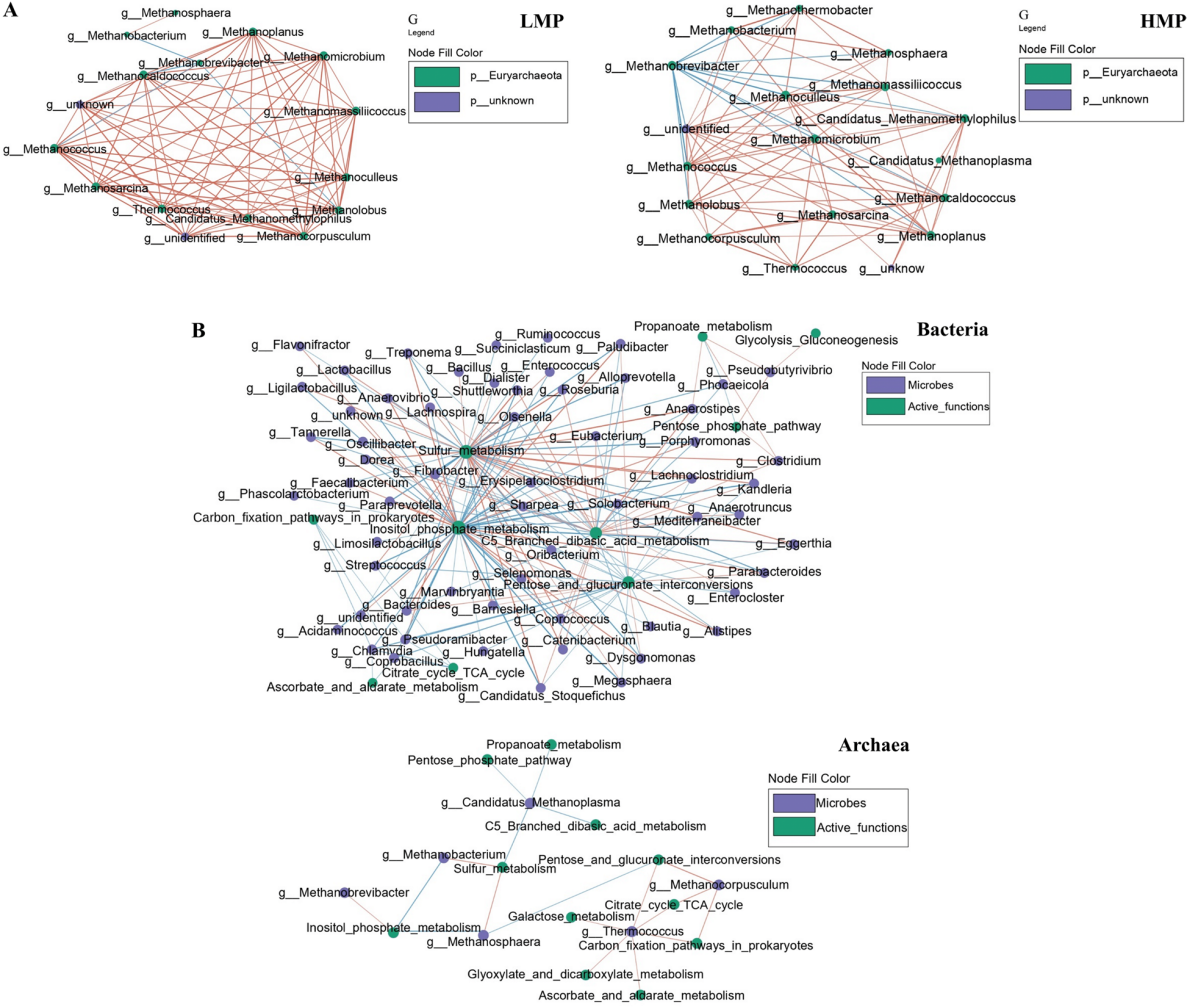
Co‑occurrence networks of microbial taxa. **A** The co‑occurrence among rumen Archaea in cows with low and high methane production. **B** Relationships between rumen microbial taxa and microbial functions. Only relationships with *P*-values less than 0.05 were shown. Positive relationships were indicated by red edges, whereas negative relationships were indicated by blue edges. Node size was proportional to the mean abundance

The co-occurrence network analysis of ruminal archaea showed 163 co-occurrence connections, with distinct patterns between the LMP and HMP groups. Eighty-two connections were found in the HMP group, and 81 connections were found in the LMP group (Fig. 7A). In addition, HMP group had 12 negative (*P* < 0.05) connections and LMP group had 4 negative (*P* < 0.05) connections, and the central archaea of these negative connections was *Methanobrevibacter* (Fig. 7A).

### Associations between microbial genera and microbial functions determine the effect of rumen bacteria and archaea on CH_4_ emissions

The co‑occurrence network revealed 76 positive and 103 negative correlations between bacterial taxa and functions (*P* < 0.05) (Fig. 7B). The “inositol phosphate metabolism” was positively (*P* < 0.05) correlated with 17 bacterial genera, including *Alistipes*, *Anaerotruncus*, *Anaerovibrio*, *Bacteroides*, *Barnesiella*, *Dysgonomonas*, *Faecalibacterium*, *Flavonifractor*, *Fibrobacter*, *Oscillibacter*, *Selenomonas*, *Tannerella*, *Treponema*, *Paludibacter*, *Parabacteroides*, *Paraprevotella*, and *Porphyromonas*; the “pentose and glucuronate interconversions” was positively (*P* < 0.05) correlated with 6 bacterial genera (*Alistipes*, *Bacteroides, Barnesiella, Dysgonomonas, Paludibacter,* and *Parabacteroides*). Meanwhile, these bacterial genera, which were positively (*P* < 0.05) related to both “pentose and glucuronate interconversions” and “inositol phosphate metabolism”, were more abundant (*P* < 0.05) in the HMP. In contrast, bacteria with higher abundance in the LMP cows, including *Lachnospira*, *Bacillus*, *Dialister*, *Olsenella*, *Roseburia*, *Eubacterium*, *Sharpea*, *Oribacterium*, *Catenibacterium*, *Clostridium*, *Lachnoclostridium*, *Kandleria*, and *Eggerthia*, were all correlated positively (*P* < 0.05) with both “sulfur metabolism” and “C5-branched dibasic acid metabolism”.

Moreover, the co‑occurrence network revealed 12 positive and 7 negative correlations between archaeal taxa and functions (*P* < 0.05) (Fig. 7B). Specifically, the archaea enriched in the HMP cows (*Methanobrevibacter*) was positively (*P* < 0.05) correlated with “inositol phosphate metabolism”, while the archaea enriched (*P* < 0.05) in the LMP (*Methanosphaera* and *Methanobacterium*) were negatively (*P* < 0.05) correlated with “inositol phosphate metabolism”. Additionally, *Candidatus Methanoplasma*, which was enriched in the HMP cows, was negatively (*P* < 0.05) associated with “sulfur metabolism”, in contrast to *Methanosphaera* and *Methanobacterium* (enriched in the LMP cows), which showed a positive (*P* < 0.05) association. In addition, both *Thermococcus* and *Methanocorpusculum* were positively (*P* < 0.05) associated with carbohydrate degradation, including “pentose and glucuronate interconversions”, “citrate cycle TCA cycle”, and “carbon fixation pathways in prokaryotes”.

### Microorganisms associated with rumen VFA profile and CH_4_ emissions

**Fig. 8:**
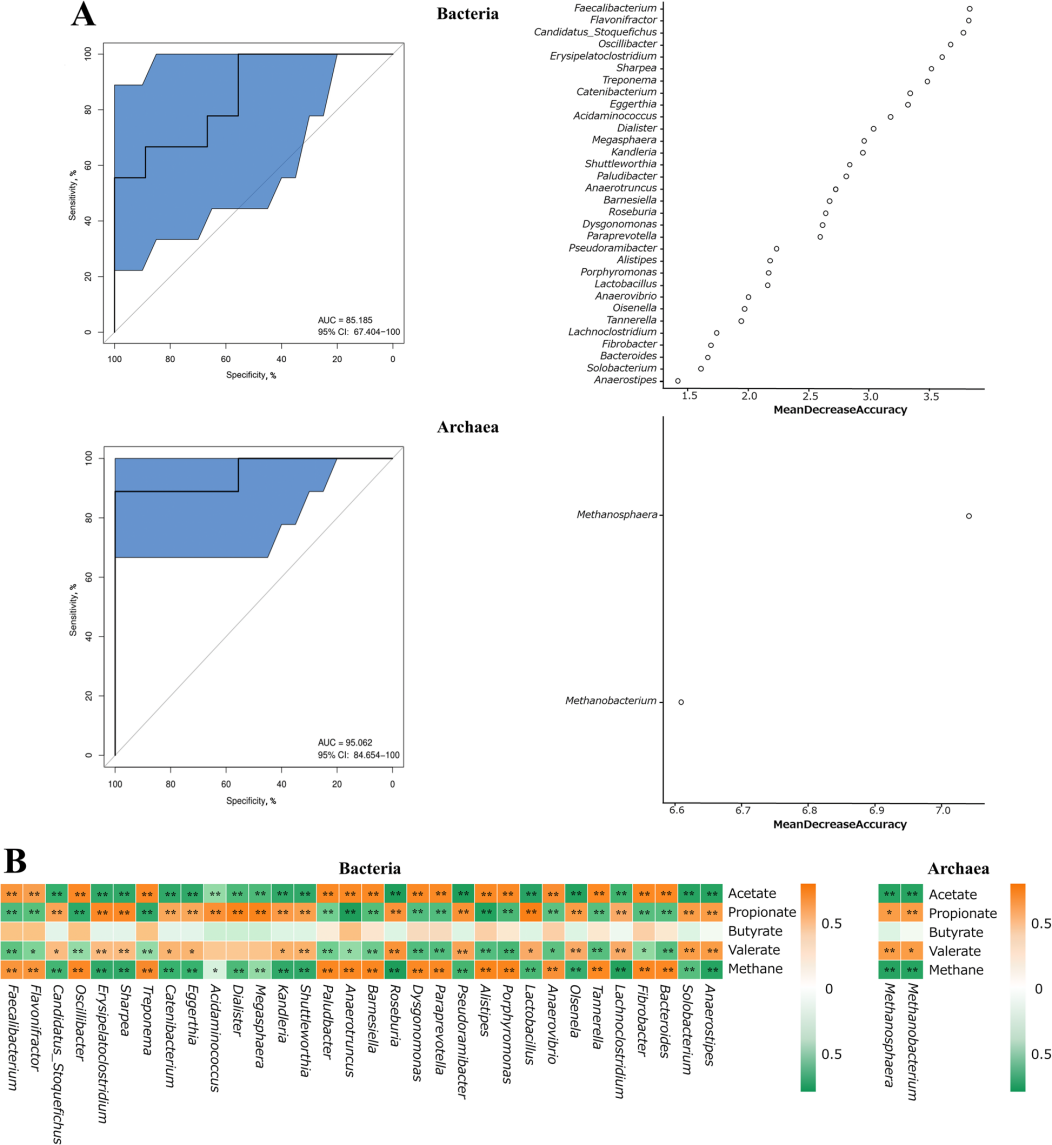
Prediction of VFA proportions and methane production using rumen microbiota. **A** Receiver operating characteristic curve and the confusion matrix for the performance of the random forest model with the selected microbiomes according to the mean decrease accuracy. **B** Spearman correlation coefficients between rumen microbiomes and proportions of VFA and methane production based on heatmaps. ^*^P < 0.05, ^**^P < 0.01

The rumen microorganisms associated with rumen VFA proportions and enteric CH_4_ production in cows were found, including microbial taxa identified as important predictors by the random forest model and statistical associations identified through correlation analysis. A total of 32 bacterial genera and 2 archaeal genera were selected as biomarkers for cows based on the mean decreasing accuracy scores in the random forest model (Fig. 8A). Fifteen bacterial genera had positive (*P* < 0.05) relationships with acetate proportion and CH_4_ production, but negative (*P* < 0.05) relationships with propionate and valerate proportions (Fig. 8B). Conversely, the remaining 17 bacterial genera had negative (*P* < 0.05) relationships with acetate proportion and CH_4_ production, while 14 of these genera had positive (*P* < 0.05) relationships with propionate and valerate proportions (Fig. 8B). Moreover, the archaeal genera *Methanosphaera* and *Methanobacterium* were negatively (*P* < 0.05) associated with acetate proportion and CH_4_ production, but positively (*P* < 0.05) associated with proportions of propionate and valerate (Fig. 8B).

## Discussion

Dairy cows are a major source of CH_4_ emissions in the livestock industry [35]. Furthermore, rumen microorganisms greatly affect or determine the CH_4_ emissions of dairy cows [36]. Understanding the mechanisms underlying inter-individual variations in rumen methanogenesis is essential to formulate CH_4_ mitigation strategies. We have integrated large-scale datasets on gas emissions from dairy cows under normal feeding conditions and characterized the natural variations in CH_4_ emissions [20]. However, the underlying rumen microbial mechanisms driving these variations remain to be elucidated. In the current study, we used metagenome analysis to investigate the rumen microbiome-dependent mechanisms underlying CH_4_ production and estimated the contributions of rumen microbial composition and functions to the variations in this trait.

### Associations between VFA metabolism and CH_4_ emissions

In general, the rumen acetate production pathway increases H_2_ and CO_2_ production, indicating that CH_4_ emissions are positively related with acetate but negatively related with propionate [37, 38]. Consistent with these findings, the HMP cows had a higher acetate proportion and lower propionate proportion in this study. Cows in the study were fed in a freestall barn with the same diet, and there was no difference in age or days in milk between the LMP and HMP, suggesting that variations in VFA and CH_4_ might be attributable to rumen microbial variation. From pyruvate to acetate, HMP cows had a higher relative abundance of glucokinase (EC:2.7.1.2), supporting a higher proportion of acetate in HMP cows. In the propionate fermentation process, 7 enzymes were abundant in HMP while only 1 enzyme (lactate dehydrogenase, EC:1.1.1.27) was abundant in the LMP. The propionate formation includes acrylate and succinate pathways, and the lactate dehydrogenase was the first step in the acrylate pathway. This result supported that the acrylate pathway may play a more prominent role in propionate formation in this study. Furthermore, *Selenomonas* and *Succiniclasticum* belonged to the Succinivibrionaceae family were enriched in HMP. These bacteria are regarded as starch-degrading bacteria that produced succinate [39], further supporting the potential dominance of the acrylate pathway. Moreover, we found that HMP cows have a higher abundance of *Butyrivibrio*, which is a formate producer that is crucial to the hydrogenotrophic methanogenesis pathway [40].

### Effect of bacteria on CH_4_ emissions

Bacteria play a great role in rumen fermentation, accounting for 95% of the total microorganisms [41]. We found considerable differences in rumen bacterial communities between cows in the LMP and HMP groups in this study. Ruminal bacterial consortiums of dairy cows were primarily composed of Bacteroides and Firmicutes, which were mainly involved in the production of hydrolytic enzymes that degrade lignocellulosic components [42]. In the current study, Bacteroides was enriched in the HMP, while Firmicutes was enriched in the LMP. Consequently, the Firmicutes/Bacteroidetes ratio was higher in the LMP relative to the HMP (1.54 vs. 1.00).

Kamke et al. [43] found that there were differences in both lactate-producing and lactate-utilizing bacteria between high- and low-methane-emission sheep. In contrast, this study revealed more pronounced bacterial differences between the HMP and LMP groups in this study. The enrichment of *Ruminococcus flavefaciens* in the HMP contributed to the difference in *Ruminococcus* genus between the two groups. *Ruminococcus flavefaciens* was considered one of the major ruminal cellulolytic bacteria, and was beneficial for H_2_ production as an acetate producer [44, 45]. Similarly, *Alistipes*, which exhibited a higher abundance in the high-forage diet group and showed a positive correlation with acetate content [46, 47], was also more abundant in the HMP cows of the present study. The degradation of ruminal starch enhanced the H_2_ flow [48], thereby promoting methanogenesis. The enrichment of starch-degrading bacteria (*Bacteroides*, *Treponema*, *Oscillibacter*, and *Treponema bryantii*) in the rumen contributed to the increased CH_4_ production observed in the HMP cows. In the current study, *Kandleria*, *Sharpea*, *Olsenella*, *Oribacterium*, *Kandleria vitulina*, and *Sharpea azabuensis* were enriched in the LMP group. Previous studies have identified these four bacterial genera and two species as producers of lactate [43, 49, 50]. Similar to lactate utilization, which competed with methanogens for H_2_ to inhibit methanogenesis, lactate production also mitigated CH_4_ production by decreasing the pH value [51]. Kamke et al. [43] found that low-methane-emission sheep converting hexoses to lactate and subsequently metabolizing it to butyrate produced 24.8% less CH_4_ than directly converting hexoses to acetate and butyrate. In this experiment, LMP was enriched with *Clostridium*, *Roseburia*, *Lachnoclostridium*, and *Megasphaera*, which were all butyrate-producing bacteria [43, 52–54]. In addition, *Catenibacterium*, a propionate-producing bacterium [55], was also enriched in the LMP cows. These supported the increased conversion of lactate to propionate and butyrate, thereby reducing CH_4_ production. Moreover, the co-occurrence networks based on rumen bacteria revealed these 3 bacterial genera (*Kandleria*, *Sharpea*, *Olsenella*) were positively related with each other but negatively correlated with *Paraprevotella*, which was enriched in the HMP (0.32% vs. 0.17%). Previous studies showed that *Paraprevotella* produces succinate and acetate [56], and exhibits a positive correlation with *Methanobrevibacter* [57]. Therefore, the negative correlations between these 3 bacterial genera and *Paraprevotella* might be attributed to competition for pyruvate. In the current study, the homoacetogens (*Eubacterium*, *Blautia*, *Eubacterium cellulosolvens*) had higher abundances in the LMP. Homoacetogenesis, as an H_2_ uptake pathway, competes with the methanogenesis for H_2_, thereby inhibiting CH_4_ production [58].

We annotated the non-redundant genes involved in the methane metabolism pathway and obtained the abundances of the corresponding bacterial and archaeal genera (Fig. S4). Consistent with the above results, the abundances of homoacetogens (*Eubacterium*, *Blautia*), lactate-producing bacteria (*Kandleria*, *Sharpea*), and lactate-utilizing bacteria (*Megasphaera*) in the LMP cows were higher than those in the HMP cows, whereas the abundance of acetogens (*Ruminococcus*, *Paraprevotella*) was lower than that in the HMP cows. The majority of the bacterial species exhibiting differential abundance between the HMP and LMP groups belonged to the *Prevotella* genus. Notably, *Prevotella ruminicola* and *Prevotella brevis*, which contribute to acetate production [59], were enriched in HMP cows. In contrast, species belonged to the *Prevotella* genus enriched in the LMP group have been associated with distinct metabolic functions: *Prevotella bryantii* produced lactate and succinate [49], *Prevotella albensis* was a starch utilizer [60], *Prevotella copri* was negatively related with CH_4_ emissions [61], *Prevotella multisacharivorax* could digest various sugars, both *Prevotella multisaccharivorax* and *Prevotella *sp.* AGR2160* contributed to the carbohydrate metabolism [62]. Therefore, Bekele et al. [63] noted that it is difficult to determine the metabolic strategy of *Prevotella* genus due to its diversity.

In summary, the comprehensive metabolic effects of starch-degrading, lactate-producing, lactate-utilizing, formate-producing, acetate-producing, propionate-producing, and butyrate-producing bacteria, as well as homoacetogens, in the metabolism of starch, lactate, and VFA contributed to the differentiation between the HMP and LMP cows. The observed bacterial variations were more pronounced than those reported in previous studies [43, 64]. This discrepancy possibly because the cows in the present study were selected from a larger herd size, and CH_4_ emissions from this herd were measured using the GreenFeed system under normal feeding conditions. Taxonomic differences of ruminal bacteria indicated that the rumen of HMP dairy cows was enriched with bacteria that provided H_2_ for methanogens, while LMP dairy cows was enriched with bacteria that competed with methanogens for H_2_.

### Contribution of archaea to CH_4_ emissions

For ruminants, all enteric CH_4_ is produced by methanogens, including *Methanobrevibacter*, *Methanosphaera*, *Methanobacterium*, and others. Similar to the findings on bacterial communities, we found more differences in archaeal composition between high and low CH_4_ emitters than previously studied [36, 64]. Specifically, *Methanobrevibacter* and *Candidatus Methanoplasma* exhibited higher relative abundances in HMP cows, whereas *Methanosphaera*, *Methanobacterium*, and *Methanothermobacter* exhibited lower ones. Overall, 10 methanogen species had higher while 14 methanogen species had lower relative abundances in HMP cows. The results of previous studies were patchy, such as Shi et al. [36] and Wallace et al. [64] only found differences in the genera *Methanobrevibacter* and *Methanosphaera*, as well as the species *Methanobrevibacter gottschalkii*, between high and low CH_4_ emitters. These results once again demonstrate the completeness and systematicity of this study.

*Methanobrevibacter* and *Methanosphaera* were the major rumen methanogens of dairy cows in this study, which was consistent with previous research [65]. Hydrogenotrophic methanogens use H_2_ + CO_2_ or formate to produce CH_4_. Among these, *Methanobrevibacter* was enriched in the HMP, while *Methanobacterium* and *Methanothermobacter* were enriched in the LMP. For methylotrophic methanogens, *Candidatus Methanoplasma* utilized methylamine and methanol to produce CH_4_ [66, 67], and *Methanosphaera* produced CH_4_ by reducing methanol using H_2_ [36]. In this study, *Candidatus Methanoplasma* was enriched in the HMP, while *Methanosphaera* was more abundant in the LMP. These findings indicated that there might be potential competition among methanogens. In addition, co-occurrence network analysis based on ruminal archaea showed that there were negative correlations among methanogens, with *Methanobrevibacter* was the center of these negative correlations. Similarly, earlier studies indicated that the relative abundance of *Methanosphaera* was negatively associated with *Methanobrevibacter* [13], as well as *Metanobacterium* competed with *Metanobrevibater* for substrates [40]. Therefore, there were negative correlations among some methanogens based on competition for substrates and spaces [17], as well as thermodynamic differences among methanogenic pathways (acetoclasty < methylotrophy < hydrogenotrophy) [68, 69].

In line with *Prevotella*, *Methanobrevibacter*, as the most abundant genus of archaea, had a total of 13 species that showed different abundances between the two groups, with 6 species enriched in the HMP and 7 species enriched in the LMP. We found species (*Methanobrevibacter smithii*, *Methanobrevibacter gottschalkii*) belonged to *Methanobrevibacter* genus enriched in the HMP group have been reported to be correlated with high CH_4_ production [22, 36], but there is little information about other archaeal species such as species belonging to the genus *Methanobrevibacter*, enriched in the LMP. Taxonomic differences of ruminal archaea suggested that simply reducing the number of one or more methanogens might not have the desired effect of mitigating enteric CH_4_ emissions due to potential compensatory competition between methanogens. In the rumen, protozoa produce large amounts of H_2_ through their hydrogenosomes and were symbiotically associated with methanogens [70]. Newbold et al. [71] indicated that eliminating rumen ciliate protozoa reduced CH_4_ production by 11% in ruminants. This study showed that in addition to Ciliophora, the 4 genera and 4 species belonged to the Ciliophora phylum were also enriched in the HMP cows (Table S1). This observation supported the higher CH_4_ production from the HMP cows.

### Effect of rumen microbial function on methanogenesis

KEGG functions analysis based on carbohydrate degradation showed that “pentose and glucuronate interconversions” and “inositol phosphate metabolism” pathways were enriched in the HMP. As a result, the HMP microorganisms might have a higher capacity to degrade carbohydrates, resulting in more hydrolytic products and pyruvate [72, 73]. Consequently, these metabolic by-products promoted the formation of CH_4_ in the HMP group. In addition, CAZymes analysis further demonstrated that the HMP were more capable of degrading complex substrates, due to genes encoding CAZymes involved in deconstructing carbohydrates (GH, CE, PL, AA, and CBM) were also enriched in the HMP. This caused the cows in the HMP group to produce more CH_4_. From the perspective of competition for H_2_, the lower CH_4_ emissions of the LMP cows may be attributed to the metabolism of H_2_-consuming compounds. Sulfate has been reported to be an electron acceptor whose reduction competes with methanogens for H_2_ [74]. On energy metabolism, the enrichment of “sulfur metabolism” pathway in the LMP provided further evidence that LMP cows produced less CH_4_ in the current study.

Meanwhile, we analyzed the methane metabolic pathway and found that HMP cows were enriched in acetyl-CoA synthetase (EC:6.2.1.1) and its corresponding gene (K01895), both of which belong to the acetoclastic pathway of methanogenesis. Similar to our results, Shi et al. [36] observed only acetyl-CoA synthetase involved in the methane metabolism pathway was enriched in high-methane-emission sheep by rumen metagenomics. The formate production pathway is crucial for rumen methanogenesis due to formate can be utilized by hydrogenotrophic methanogens to produce CH_4_. Within the process of converting pyruvate to formate and subsequently to CO_2_ and H_2_, both dihydrolipoyllysine-residue acetyltransferase (EC:2.3.1.12) and formate dehydrogenase (EC:1.2.1.2), along with their respective genes (K00627 and K00123), were found to be enriched in HMP cows in the present study (Table S7). However, Shi et al. [36] did not find similar results in rumen metagenomic analysis. Among the three methanogenic pathways, the enrichment of both the formate production pathway (belonging to hydrogenotrophy) and the acetoclastic pathway also led to higher CH_4_ production in the HMP cows.

In addition to being converted to VFA and CH_4_, pyruvate is also converted to valine, leucine, and isoleucine. Thus, amino acid metabolism indirectly affects CH_4_ production. The “valine, leucine and isoleucine biosynthesis” involved in amino acid metabolism was enriched in the LMP cows, supporting that the LMP cows produced less CH_4_. Branched-chain amino acids such as valine, leucine, and isoleucine are important contributors to rumen microbial proteins, which may be beneficial to milk protein synthesis of cows [75]. In addition to “valine, leucine and isoleucine biosynthesis”, “C5-branched dibasic acid metabolism” was also enriched in the LMP cows. Previous studies reported that “C5-branched dibasic acid metabolism” was affected by nitrogen content and positively correlated with “valine, leucine and isoleucine biosynthesis” [76, 77], which is consistent with this study.

Furthermore, co‑occurrence networks based on bacterial genera and bacterial functions showed that, bacteria with higher abundance in the HMP cows were positively correlated with both “pentose and glucuronate interconversions” and “inositol phosphate metabolism”, while bacteria with higher abundance in the LMP cows were positively correlated with both “sulfur metabolism” and “C5-branched dibasic acid metabolism”. Co‑occurrence network based on archaeal genera and archaeal functions also showed that, archaea with higher abundance in the HMP cows was positively related with “inositol phosphate metabolism” but negatively related with “sulfur metabolism”. In the HMP group, although cows might obtain more pyruvate due to the advantage in carbohydrate degradation, a greater proportion of this pyruvate was converted to CH_4_, while a smaller fraction was utilized for branched-chain amino acids synthesis.

### Microbial biomarkers of CH_4_ emissions

In the present study, we used random forest analysis to obtain biomarkers of cows, and explored the relationships between these biomarkers and cow phenotypes. Correlation analysis further revealed that a total of 34 genera had significant relationships with VFA proportions and CH_4_ production of dairy cows. Microorganisms (15 bacterial genera) positively related to acetate proportion and CH_4_ production were enriched in the HMP cows, while other microorganisms (17 bacterial genera and 2 archaeal genera) were enriched in the LMP cows. These rumen microbial biomarkers can be used to evaluate CH_4_ production from dairy cows. Synthetically, the deep metagenomic sequencing of rumen contents in this study revealed that variation of CH_4_ production between groups was determined by microbes and the enzymes and genes of methanogenic pathways.

## Conclusion

Based on the empirically measured CH_4_ emissions and rumen metagenomics analysis of dairy cows, the following conclusions can be drawn: (1) Under the same environmental and feeding conditions, there was a huge difference in CH_4_ emissions of dairy cows due to rumen microbiome and physiological nutrition reasons. (2) The difference in CH_4_ emissions between the HMP and LMP cows was associated with the integrated regulation of bacteria, archaea, and protozoa, not a single microbial type. For instance, bacterial metabolizers of VFA, lactate, and starch, as well as homoacetogens, all act as regulators of methane production. Notably, co-occurrence network analysis of the rumen microbiota uncovered there were negative associations among methanogens, with *Methanobrevibacter* serving as a central node in these associations. Simply reducing the population of certain methanogens may not achieve the desired effect of mitigating CH_4_ emissions. (3) Among the nutrient metabolic pathways, 4 pathways (“valine, leucine and isoleucine degradation”, “lysine degradation”, “pentose and glucuronate interconversions”, “inositol phosphate metabolism”) were positively associated with CH_4_ emissions and 3 pathways (“valine, leucine and isoleucine biosynthesis”, “C5-branched dibasic acid metabolism”, “sulfur metabolism”) were negatively associated with CH_4_ emissions. (4) In the methanogenic pathways, formate metabolism and acetoclastic pathways were characteristic features of the HMP cows exhibiting high CH_4_ emissions. The findings of the current study provide novel insights into the regulation of rumen methanogenesis, as well as valuable data for breeding and selection of cows with low CH_4_ production. Compared with previous studies, it provided a more systematic and detailed perspective for advancing low-carbon dairy farming.

## Supplementary Information

Additional file 1: Table S1. Rumen microbial taxa identified in the metagenomes. Table S2. Enzymes involved in the methane metabolism pathway in cows. Table S3. Genes involved in the methane metabolism pathway in cows. Table S4. The alpha-diversity of rumen microbial community. Table S5. KEGG level-3 pathways identified in the metagenomes. Table S6. CAZymes composition according to the enzymes at class and family level. Table S7. Enzymes and genes related to formate metabolic pathway. 

Additional file 2: Fig. S1. Comparison of microbial domains between cows with low and high methane production.

Additional file 3: Fig. S2. Differential CAZyme functions between cows with low and high methane production.

Additional file 4: Fig. S3. Co‑occurrence of rumen bacteria in cows with low and high methane production. 

Additional file 5: Fig. S4. Relative abundances of bacterial and archaeal communities involved in methane metabolism at the genus level.

## Data Availability

All data generated or analyzed during this study were included in this published article and its Supplementary Information files. The rumen metagenome sequences were deposited into NCBI Sequence Read Archive (SRA) under the accession number of PRJNA980404.

## Abbreviations

CBMs: Carbohydrate-binding module
GHG: Greenhouse gas
GTs: Glycosyltransferases
HMP: High-methane-producing cows
KEGG: Kyoto Encyclopedia of Genes and Genomes
LMP: Low-methane-producing cows
VFA: Total volatile fatty acid
