# Supplementary figures and images for: Variations in methane emissions from dairy cows: associations with rumen microbial synergy and metabolic pathway divergence
Source: J Anim Sci Biotechnol. 2026 Jun 15;17:121. doi: 10.1186/s40104-026-01432-9 (PMC13267709; doi:10.1186/s40104-026-01432-9)

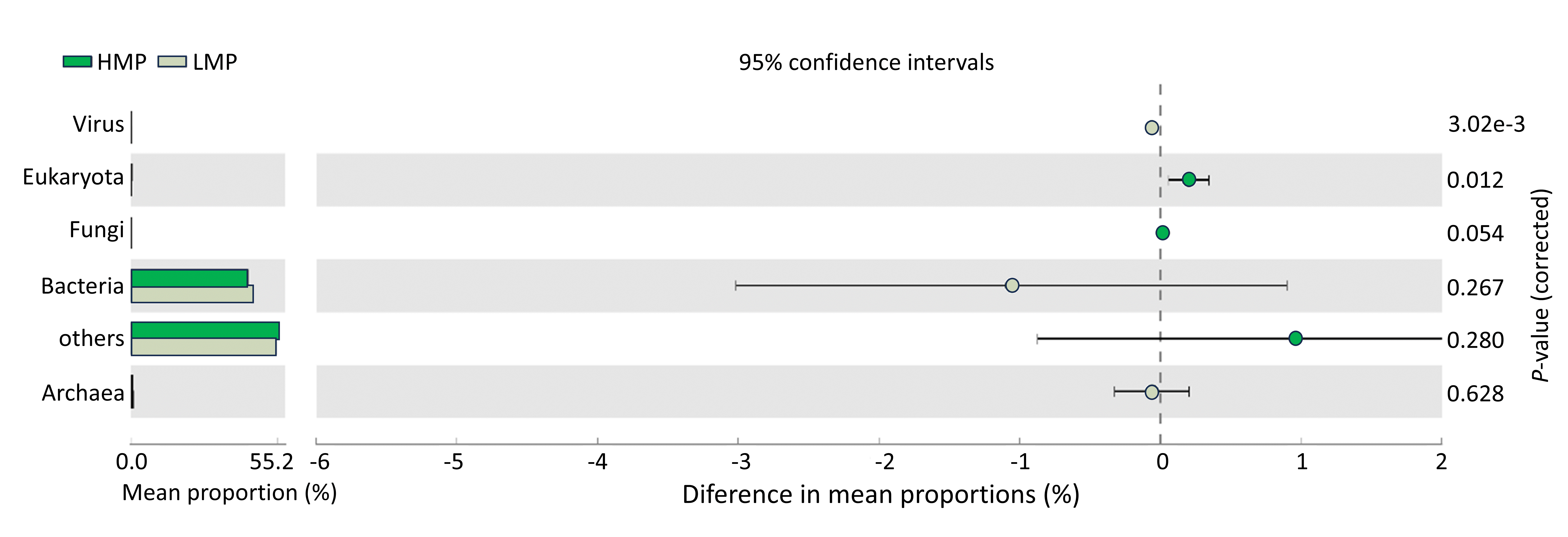

Supplement: Supplementary file 2 — Additional file 2: Fig. S1. Comparison of microbial domains between cows with low and high methane production. [file 40104_2026_1432_MOESM2_ESM.gif]

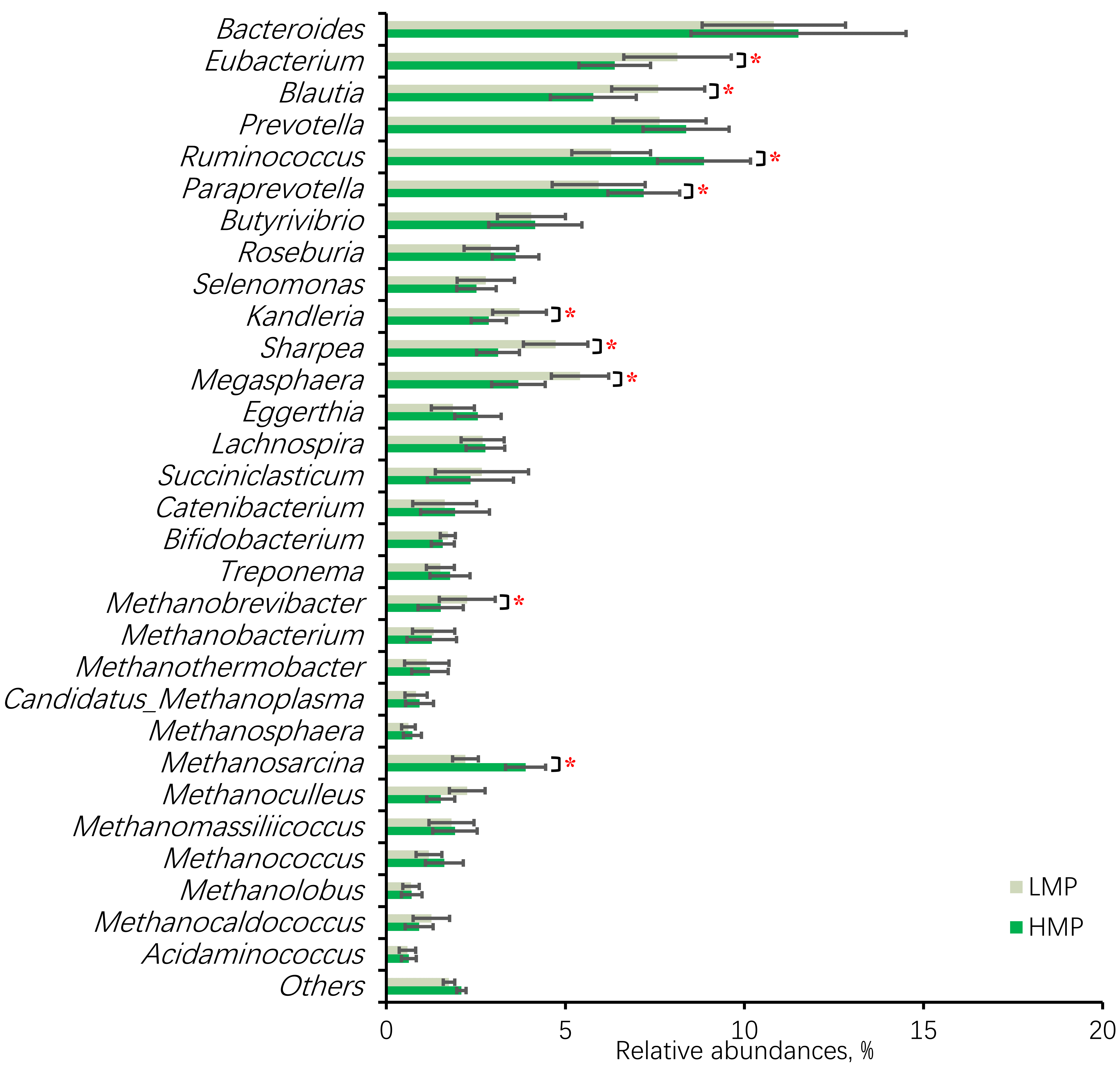

Supplement: Supplementary file 5 — Additional file 5: Fig. S4. Relative abundances of bacterial and archaeal communities involved in methane metabolism at the genus level. [file 40104_2026_1432_MOESM5_ESM.gif]
